# A Case of Buccal Clear Cell Carcinoma Caused by Rare Fusion Gene: *EWSR1-CREM*

**DOI:** 10.1155/2021/5557247

**Published:** 2021-10-20

**Authors:** Miyako Hoshino, Katsuyuki Inoue, Tomohisa Kaneda, Michiko Nishimura, Kaoru Kusama, Hideaki Sakashita, Yukiko Sato, Kengo Takeuchi, Toshitaka Nagao, Kentaro Kikuchi

**Affiliations:** ^1^Division of Pathology, Department of Diagnostic and Therapeutic Sciences, Meikai University School of Dentistry, 1-1 Keyakidai, Sakado, Saitama 350-0283, Japan; ^2^Second Division of Oral and Maxillofacial Surgery, Department of Diagnostic and Therapeutic Sciences, Meikai University School of Dentistry, 1-1 Keyakidai, Sakado, Saitama 350-0283, Japan; ^3^Division of Pathology, Cancer Institute, Japanese Foundation for Cancer Research, 3-8-31 Ariake, Koto-ku, Tokyo 135-8550, Japan; ^4^Department of Pathology, Cancer Institute Hospital, Japanese Foundation for Cancer Research, 3-8-31 Ariake, Koto-ku, Tokyo 135-8550, Japan; ^5^Pathology Project for Molecular Targets, Cancer Institute, Japanese Foundation for Cancer Research, 3-8-31 Ariake, Koto-ku, Tokyo 135-8550, Japan; ^6^Department of Anatomic Pathology, Tokyo Medical University, 7-3-1 Hongo, Bunkyo-ku, Tokyo 113-0033, Japan

## Abstract

Clear cell carcinoma (CCC) is a rare entity in the salivary gland tumor. So far, only 10 cases of primary CCC of the buccal mucosa have been reported. Here, we first report an extremely rare case of buccal CCC with the *EWSR1-CREM* fusion gene. The patient, a 69-year-old woman, presented with a painless mass in the right buccal mucosa. The tumor, which had been present for about 10 years, measured approximately 15 mm in diameter and was pedunculated, elastic hard, smooth, and mobile. Histopathological examination revealed proliferating tumor cells with vacuolated and clear cytoplasm partially surrounded by hyalinized stroma. The tumor was not encapsulated, and no contact with the overlying epithelium was evident. Duct-like structures were occasionally observed in the tumor nests composed of clear cells. The tumor had invaded into surrounding muscle and adipose tissues. Immunohistochemical examination revealed that the clear cells were positive for epithelial cell markers, and myoepithelial markers were negative. Fluorescence in situ hybridization (FISH), performed to search for genetic abnormalities, demonstrated split positivity for *EWSR1*, and fusion with *CREM* was confirmed. These findings suggested a diagnosis of CCC.

## 1. Introduction

Clear cell carcinoma (CCC) is an epithelial malignant tumor that arises in the salivary glands. CCC in the salivary glands was first reported in 1994 by Milchgrub et al. [1], who described a low-grade salivary gland carcinoma composed of malignant cells with clear cytoplasm, with hyalinization. CCC has a squamoid phenotype and lacks certain features of other clear cell-rich salivary gland carcinomas. It is more common in women, typically presenting in the fifth to eighth decades of life, and rare in children [2]. CCC is rare and accounts for fewer than 1% of all malignant tumors in the salivary glands. It occurs most frequently in the intraoral minor salivary glands, the palate, and the base of the tongue and less frequently in the buccal mucosa [3]. CCC most commonly presents as swelling and may be ulcerated or associated with pain, bone invasion, and soft tissue fixation [2]. Histopathologically, CCC is composed of proliferating epithelial cells with clear cytoplasm organized into trabeculae, cords, or solid nests surrounded by hyalinized stroma [1]. However, differential diagnosis can be difficult because the microscopic features of CCC frequently overlap with those of other salivary gland tumors and metastatic renal cell carcinoma [3]. *EWSR1-ATF1* fusion specific for CCC can be evaluated by RT-PCR as hallmark markers, but in our case, *EWSR1-ATF1* fusion was not detected. To our knowledge there are previously 3 reported cases of hyalinizing clear cell carcinomas of *EWSR1-CREM* fusion [4]; as an additional case report in the literature, here, we first report a very rare case of buccal CCC with the *EWSR1-CREM* fusion gene.

## 2. Case Report

A 69-year-old woman presented with a mass in the right buccal mucosa that had been present for about 10 years and untreated because of absence of any pain.

**Figure 1:**
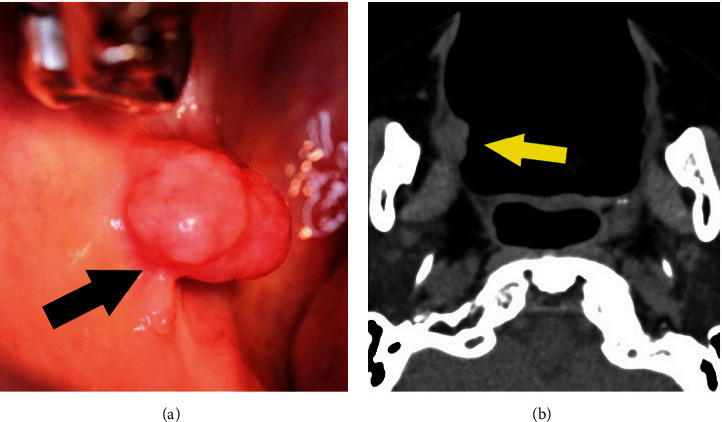
Intraoral appearance of the buccal mucosa and CT image. (a) The mucosal epithelium was normal in color with a smooth surface. There was no induration around the tumor (black arrow). (b) CT (axial view) showed an enhanced lesion on the anterior portion of the right palatoglossal arch (yellow arrow).

**Figure 2:**
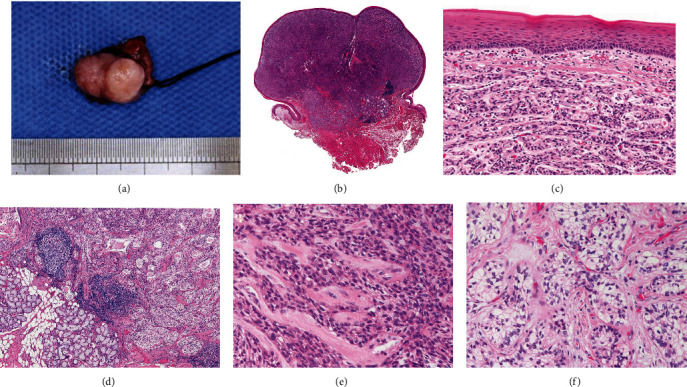
Microscopic and survey view of the surgical resected specimen. (a) Resected specimen measuring 18 × 12 × 14 mm. (b, c) The tumor demonstrated proliferation of tumor nests with a fibrous stroma. There was no capsule and no contact with the covering epithelium. (d) The tumor had invaded into surrounding muscle and adipose tissues. (e, f) The tumor nests were composed of epithelioid and clear cells partially surrounded by hyalinized stroma. Features suggestive of transition between the two cell types were observed (HE; original magnification: (b) ×1, (c) ×20, (d, f) ×40, and (e) ×100).

However, as it showed no tendency to improve, and the swelling worsened, she visited a local dentist at first. She was then referred to the Meikai University Hospital for detailed examination and treatment. At the first visit, examination of the oral cavity demonstrated a mass measuring 15 × 10 mm in the right buccal mucosa on the anterior portion of the lobular polypoid palatoglossal arch (Figure 1(a)). The mucosal epithelium was normal in color with a smooth surface. There was no induration around the tumor. CT showed an enhanced lesion on the anterior portion of the right palatoglossal arch (Figure 1(b)). Surgical excision of the tumor was performed under local anesthesia. The surgical specimen measured 18 × 12 × 14 mm (Figure 2(a)). Histopathologically, the tumor showed proliferation of tumor nests with fibrous stroma. There was no capsule and no contact with the covering epithelium (Figures 2(b) and 2(c)). The tumor invaded surrounding structures such as muscle and adipose tissue (Figure 2(d)).

The tumor nests were composed of epithelioid and clear cells with partially hyalinized stroma (Figures 2(e) and 2(f)). Mitotic figures were rarely evident, and transition between the two cell types was observed in part (data not shown).

**Figure 3:**
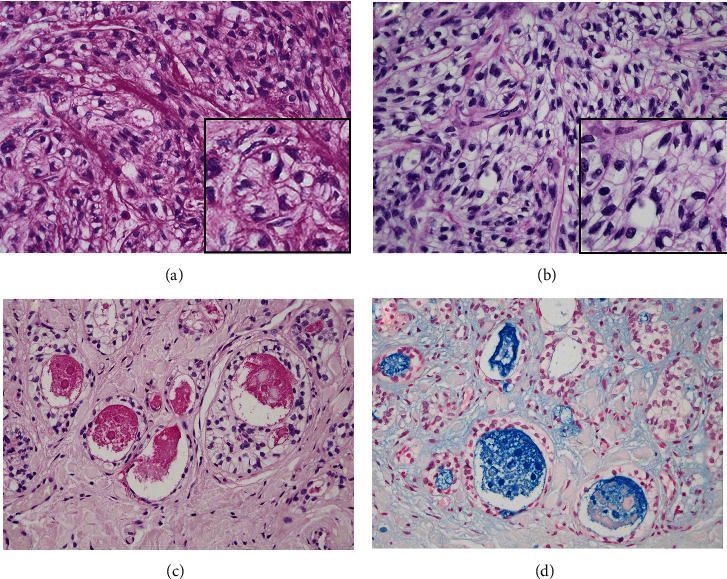
Special stains: (a) PAS, (b) d-PAS, (c) mucicarmine, and (d) Alcian blue. PAS stain shows positive cytoplasmic red material in the clear cells, but PAS with diastase stain was negative, suggesting the presence of glycogen. The contents of duct-like structures were positively stained by mucicarmine and Alcian blue, but mucus-producing cells were not evident in the tumor nests ((a–d) original magnification ×200).

**Figure 4:**
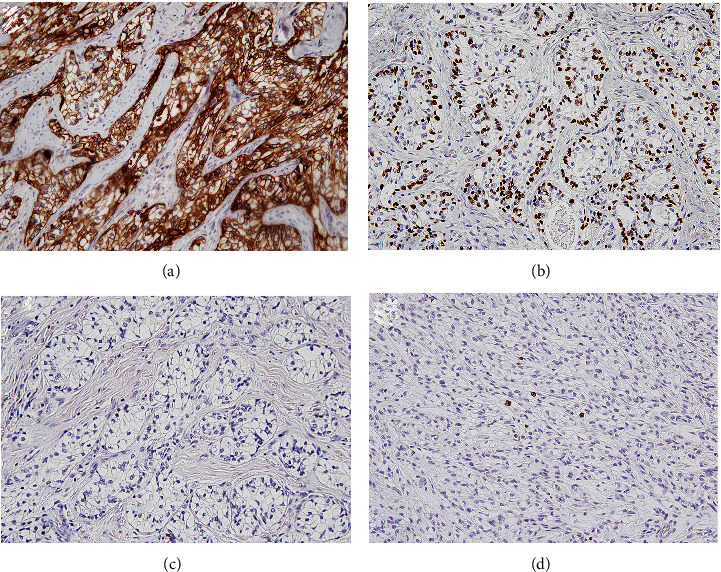
Immunohistochemical findings: (a) pancytokeratin (AE1/AE3), (b) p63, (c) S100, and (d) Ki-67. The tumor cells were stained intensely with cytokeratins, but not myoepithelial markers. The nuclei of the clear cells were positive for p63. Ki-67-positive cells were rarely found in the tumor nests ((a–d) original magnification ×200).

**Figure 5:**
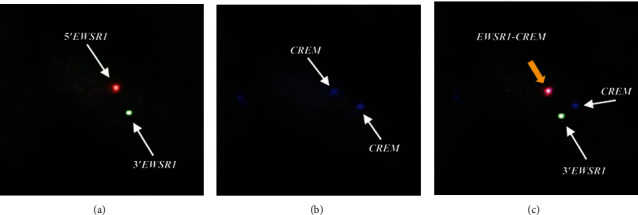
FISH analysis: (a) *EWSR1* break-apart; (b) *CREM*; (c) *EWSR1-CREM* gene fusion. *EWSR1* break-apart FISH demonstrate a cell with split red and green signals, indicative of a translocation. This case demonstrated split positivity for *EWSR1*, and fusion with *CREM* was confirmed.

Intracytoplasmic fine granules were positive for periodic acid-Schiff (PAS) and were negative for diastase-sensitive PAS (Figures 3(a) and 3(b)). The contents of duct-like structures were positively stained by mucicarmine and Alcian blue, but mucus-producing cells were not evident in the tumor nests (Figures 3(c) and 3(d)). Immunohistochemically, the tumor cells were strongly positive for AE1/AE3 (Figure 4(a)), CK5/6, and p63 (Figure 4(b)), but negative for *α*-SMA and S100 (Figure 4(c)), and Ki-67-positive cells were rarely found in the tumor nests (Figure 4(d)). The *EWSR1-ATF1* fusion gene could not be detected by reverse transcription polymerase chain reaction (RT-PCR) in the present case. RT-PCR also failed to detect the *CRTC1/3-MAML2* fusion gene, which is specific for mucoepidermoid carcinoma. Using bacterial artificial chromosome clone-derived DNA probes for *EWSR1* and *CREM* (the names of BAC clones used will be provided upon request), fluorescence in situ hybridization (FISH) analysis revealed the presence of *EWSR1-CREM* (Figures 5(a)–5(c)). These findings suggested a diagnosis of CCC with *CREM* fusion instead of *ATF1* fusion.

## 3. Discussion

CCC is a low-grade malignancy with a good prognosis after complete surgical excision. Local recurrence and nodal metastases may occur, but distant metastasis and death due to disease are rare [2]. Clinical features, staining patterns, fusion type, pathological findings, and diagnostic methods reported cases of CCC in the cheek or buccal mucosa are summarized in Tables 1 and 2. The *EWSR1-CREM* fusion gene has not been previously reported in CCC of the buccal mucosa. Differential diagnosis of CCC based on histopathology includes mucoepidermoid carcinoma, acinic cell carcinoma, clear cell oncocytoma, epithelial myoepithelial carcinoma, malignant myoepithelioma, and metastatic renal cell carcinoma, all of which show a significant proportion of clear cells [1, 5]. The clear cell variant of mucoepidermoid carcinoma is positively stained with mucicarmine for tumor cells, which can be very helpful for highlighting the mucous cells and distinguishing them from other clear cell neoplasms [6]. Acinic cell carcinoma contains zymogen granules, which are PAS-positive and diastase resistant [7]. DOG1 and SOX10 are immunopositive in acinar and intercalated duct cells. Acinic cell carcinoma is usually immunonegative for mammaglobin [8]. Clear cell oncocytoma has a PAS-positive and diastase-PAS-negative cytoplasm. Unlike CCC, it is encapsulated or circumscribed, and the clear cells have a marginal rim of cytoplasm that retains eosinophilic granules. The tumor cells are also positively stained with phosphotungstic acid-hematoxylin and show strong immunohistochemical staining for mitochondria [9]. Epithelial myoepithelial carcinoma and malignant myoepithelioma express S100 protein, *α*-smooth muscle actin (*α*-SMA), and SOX10 [6, 10]. SOX10 is positive for myoepithelial carcinoma [10]. Renal cell carcinoma shows immunopositivity for cytokeratins, vimentin, and CD10 [6]. CCC is characterized by trabeculae, cords, or irregular solid nests surrounded by a hyalinized stroma. The tumor cells have clear cytoplasm and circular or polygonal nuclei [11]. Perineural and bone invasion are common, and ducts and gland-like spaces may be evident. CCC is positive for CKs and p63 and negative for other myoepithelial markers. Intracytoplasmic fine granules that give a diastase-sensitive positive PAS reaction are present [2]. The use of special stains and immunohistochemistry, along with careful histological examination of the tumor, in order to identify the typical features found in each of these neoplasms, is helpful for establishing a correct diagnosis [5]. Specific gene fusions have been found to play a definitive role in tumorigenesis. The 2017 WHO classification states that the *EWSR1*-*ATF1* fusion gene can be identified in CCC [2] and is the most reliable tool for differentiating CCC from other histologically similar tumors. In the present case, however, it could not be detected by RT-PCR. Furthermore, the *CRTC1/3-MAML2* fusion gene characteristic of mucoepidermoid carcinoma was not detected. FISH, performed to search for genetic abnormalities, demonstrated split positivity for *EWSR1*, and fusion with the cAMP response element modulator (*CREM*) was confirmed. Recurrent gene fusions involving *EWSR1* with members of the cAMP response element binding protein (*CREB*) family (*ATF1* and *CREB1*) have been reported in a diverse group of tumors including angiomatoid fibrous histiocytoma, soft tissue and gastrointestinal clear cell sarcoma, primary pulmonary myxoid sarcoma, and hyalinizing clear cell carcinoma of the salivary gland [12]. *CREB* can bind to a few thousand gene promoters that contain *CRE* (cAMP response element), although it remains unknown what fraction of these genes is also functionally regulated upon this binding. *CREM* belongs to the *CREB* family (*ATF1*, *CREB1*, and *CREM*) of transcription factors [12]. The structure and biological functions of both *CREM* and *ATF-1* are similar to *CREB*, which forms heterodimers with *ATF-1* or *CREM* [13]. In the present case, *EWSR1* was fused with one of the *CREB* family members, *CREM*. To our knowledge, there are 3 reported cases of hyalinizing clear cell carcinomas of *EWSR1-CREM* fusion [4], but it has not been reported previously in buccal mucosa. The present case of CCC shows that *CREM* may replace *ATF1* as an *EWSR 1* fusion partner. For future characterization of tumors, molecular diagnostic assays will become increasingly important in addition to the use of special staining and immunohistochemistry.

## 4. Conclusion

CCC is a rare minor salivary gland tumor exhibiting low-grade malignancy. The diagnosis of clear cells in salivary glands can be challenging and the differential diagnosis being quite broad. However, testing for any *EWSR1* translocation combined with specific histological staining may be a new reliable method for distinguishing CCC from other salivary gland tumors. Here, we have reported an extremely rare case of clear cell-rich salivary gland carcinoma of the buccal mucosa.

## Figures and Tables

**Table 1:** Clinical features and staining patterns of previously reported cheek or buccal mucosa CCC cases.

Authors	Year	Age	Sex	Size (cm)	Metastasis	Immunohistochemical staining						Special staining				Fusion type	Treatment	Recurrence	Follow-up and outcome
						CK	p63	CD10	S100	*α*-SMA	Ki-67	PAS	d-PAS	MC	ALC				
Simpson et al. [14]	1990	66	M	2.5	—	+	NA	NA	—	NA	NA	NA	NA	NA	NA	NA	Sur	—	NED (11 y)
Hiroki et al. [15]	1999	30	F	3	—	+	NA	NA	—	NA	NA	+	NA	NA	—	NA	Sur	—	NED
Milchgrub et al. [16]	2000	29	F	2	+^†^	NA	NA	NA	NA	NA	NA	NA	NA	NA	NA	NA	Sur	+	REC (12 mo)
O'Sullivan-Mejia et al. [17]	2009	78	F	1.7	—	+	—	NA	NA	NA	NA	+	NA	—	NA	NA	Sur	—	NED
Solar et al. [9]	2009	49	F	3	NA	+	NA	NA	—	—	NA	+	—	—	NA	NA	Sur	+	REC × 2 (114 mo)
Onoda et al. [11]	2012	59	F	0.8	—	+	—	—	—	—	NA	+	—	NA	—	NA	Sur	—	NED (4 y)
Shah et al. [18]	2013	46	F	NA	+^†^	+	+	NA	—	—	NA	NA	NA	NA	NA	*EWSR1 re*	Rad, RS	+	NA
	2013	37	F	NA	+^†^	+	+	NA	—	—	NA	NA	NA	NA	NA	*EWSR1 re*	Sur	+	NA
Yamanishi et al. [3]	2015	52	F	1.5	—	+	+	NA	—	—	NA	NA	NA	—	NA	*EWSR1-ATF1*	Sur	—	NED (11 y)
Albergotti et al. [19]	2016	70	F	4.9	—	NA	NA	NA	NA	NA	NA	NA	NA	NA	NA	*EWSR1 re*	Sur	—	NED (13 mo)
Hernandez-Prera et al. [20]	2017	57	F	2	—	NA	+	NA	—	—	NA	NA	NA	—	NA	*EWSR1-ATF1*	Sur	—	NED (24 mo)
Present study	2020	69	F	1.5	—	+	+	—	—	—	±	+	—	—	—	*EWSR1-CREM*	Sur	—	NED (2 y)

NA: not available or not mentioned; NED: no evidence of disease; REC: recurrence; MC: mucicarmine; ALC: Alcian blue; *re*: rearrangement; Rad: radiotherapy; RS: reduction surgery; Sur: surgery. ^†^Cervical lymph nodes.

**Table 2:** Pathological findings and diagnostic methods of previously reported cheek or buccal mucosa CCC cases.

Authors	Findings						Diagnosis
	Solid sheets	Nests	Cords	Trabeculae	Hyalinizing stroma	Ducts and gland-like spaces	
Simpson et al. [14]	+	+	+	NA	+	NA	IHC, EMS
T Hiroki [15]	+	NA	NA	NA	+	+	IHC, EMS
Milchgrub et al. [16]	NA	+	+	+	+	NA	FNA
O'Sullivan-Mejia et al. [17]	NA	+	+	+	+	NA	IHC
Solar et al. [9]	+	+	+	+	+	NA	IHC
Onoda et al. [11]	+	NA	+	NA	+	NA	IHC
Shah et al. [18]	+	+	NA	+	+	NA	IHC, FISH
	+	+	NA	+	+	NA	IHC, FISH
Yamanishi et al. [3]	+	+	+	+	+	NA	IHC, RT-PCR
Albergotti et al. [19]	NA	+	+	NA	+	NA	FISH
Hernandez-Prera [20]	+	+	NA	+	+	NA	IHC, FISH
Present study	+	+	+	+	+	+	IHC, FISH

## References

[1] Milchgrub S., Gnepp D. R., Vuitch F., Delgado R., Albores-Saavedra J. (1994). Hyalinizing clear cell carcinoma of salivary gland. *American Journal of Surgical Pathology*.

[2] Wenig B. M., Bell D., Chiosea S., Inagaki H., Seethala R., El-Naggar A. K., Chan J. K. C., Grandis J. R., Takata T., Slootweg P. J. (2017). Clear cell carcinoma. *World Health Organization Classification of Head and Neck Tumours*.

[3] Yamanishi T., Kutsuma K., Masuyama K. (2015). A case of hyalinizing clear cell carcinoma, so-called clear cell carcinoma, not otherwise specified, of the minor salivary glands of the buccal mucosa. *Case Reports in Otolaryngology*.

[4] Chapman E., Skalova A., Ptakova N. (2018). Molecular profiling of hyalinizing clear cell carcinomas revealed a subset of tumors harboring a novel *EWSR1*-*CREM* fusion. *American Journal of Surgical Pathology*.

[5] Chao T. K., Tsai C. C., Yeh S. Y., Teh J. E. (2004). Hyalinizing clear cell carcinoma of the hard palate. *The Journal of Laryngology & Otology*.

[6] Maiorano E., Altini M., Favia G. (1997). Clear cell tumors of the salivary glands, jaws, and oral mucosa. *Seminars Diagnostic Pathology*.

[7] Pujary K., Rangarajan S., Nayak D. R., Balakrishnan R., Ramakrishnan V. (2008). Hyalinizing clear cell carcinoma of the base of tongue. *International Journal of Oral and Maxillofacial Surgery*.

[8] Simpson R. H. W., Chiosea S., Katabi N., Leivo I., Vielh P., Williams M. D., EI-Naggar A. K., Chan J. K. C., Grandis J. R., Takata T., Slootweg P. J. (2017). Acinic cell carcinoma. *World Health Organization Classification of Head and Neck Tumours*.

[9] Solar A. A., Schmidt B. L., Jordan R. C. K. (2009). Hyalinizing clear cell carcinoma. *Cancer*.

[10] Hsieh M. S., Lee Y. H., Chang Y. L. (2016). SOX10-positive salivary gland tumors: a growing list, including mammary analogue secretory carcinoma of the salivary gland, sialoblastoma, low-grade salivary duct carcinoma, basal cell adenoma/adenocarcinoma, and a subgroup of mucoepidermoid carcinoma. *Human Pathology*.

[11] Onoda M., Seki K., Ikari T., Kumamaru W., Sugiura T., Shirasuna K. (2012). A case of clear cell carcinoma, not otherwise specified (NOS) of the buccal mucosa. *Japanese Journal of Oral and Maxillofacial Surgery*.

[12] Kao Y. C., Sung Y. S., Zhang L. (2017). *EWSR1* fusions with *CREB* family transcription factors define a novel myxoid mesenchymal tumor with predilection for intracranial location. *American Journal of Surgical Pathology*.

[13] Wu X., Spiro C., Owen W. G., McMurray C. T. (1998). cAMP Response Element-binding Protein Monomers Cooperatively Assemble to Form Dimers on DNA. *Journal of Biological Chemistry*.

[14] Simpson R. H. W., Sarsfield P. T. L., Clarke T., Babajews A. V. (1990). Clear cell carcinoma of minor salivary glands. *Histopathology*.

[15] Toyoda H., Yamaguchi K., Masumoto K., Koyama T., Fukuda H., Hashimoto K. (1999). A case of hyalinizing clear cell carcinoma of the cheek with thirteen-year delay of the disease. *Journal of the Japanese Stomatological Society*.

[16] Milchgrub S., Vuitch F., Hossein Saboorian M., Hameed A., Wu H., Albores-Saavedra J. (2000). Hyalinizing clear-cell carcinoma of salivary glands in fine-needle aspiration. *Diagnostic Cytopathology*.

[17] O’Sullivan-Mejia E. D., Massey H. D., Faquin W. C., Powers C. N. (2009). Hyalinizing clear cell carcinoma: report of eight cases and a review of literature. *Head and Neck Pathology*.

[18] Shah A. A., LeGallo R. D., van Zante A. (2013). *EWSR1* genetic rearrangements in salivary gland tumors. *American Journal of Surgical Pathology*.

[19] Albergotti W. G., Bilodeau E. A., Byrd J. K., Mims M. M., Lee S., Kim S. (2016). Hyalinizing clear cell carcinoma of the head and neck: case series and update. *Head & Neck*.

[20] Hernandez–Prera J. C., Kwan R., Tripodi J. (2017). Reappraising hyalinizing clear cell carcinoma: a population-based study with molecular confirmation. *Head & Neck*.

